# Application of Brain–Computer Interface Technology in Vascular Cognitive Impairment: A Systematic Review

**DOI:** 10.3390/brainsci16060589

**Published:** 2026-05-29

**Authors:** Jieyang He, Tingting Mao, Yubiao Sun, Ling Guan

**Affiliations:** 1 Department of Biomedical Engineering, School of Medical Science and Engineering, Beijing Institute of Technology, Beijing 100081, China; 2 School of Interdisciplinary Science, Beijing Institute of Technology, Beijing 100081, China; 3 Department of Neurology, Beijing Tiantan Hospital, Capital Medical University, Beijing 100070, China; 4 Department of Medicine, The University of British Columbia, Vancouver, BC V6T 1Z4, Canada

**Keywords:** brain–computer interface, neurofeedback, electroencephalography, cognitive function

## Abstract

**Highlights:**

**What are the main findings?**

- Brain–computer interface (BCI)-based approaches show some efficacy for objective cognitive assessment and are associated with improvements in attention, memory, executive function and language in patients with vascular cognitive impairment (VCI), especially in patients with stroke.
- Substantial heterogeneity and limited mechanistic understanding highlight the need for standardized protocols and well-designed studies to support clinical translation.

**What are the implications of the main findings?**

- BCI-based approaches have the potential to complement traditional paper-and-pencil assessments by providing objective cognitive evaluation, and may serve as a promising adjunct tool for cognitive rehabilitation in patients with VCI.
- Future studies integrating multimodal neuroimaging and rigorously designed clinical trials are needed to elucidate the mechanisms underlying BCI-mediated cognitive improvement and to optimize their clinical application.

**Abstract:**

**Background:** Vascular cognitive impairment (VCI) is a common consequence of cerebrovascular diseases and significantly affects multiple cognitive domains. Brain–computer interface (BCI) technology has emerged as a promising tool for cognitive assessment and rehabilitation in patients with VCI. This systematic review examined the current applications of BCI technology for cognitive function in patients with VCI. **Methods:** In accordance with the PRISMA 2020 guidelines, we searched Medline, PubMed, Web of Science, Embase, and the Cochrane Central Register of Controlled Trials. We included studies published between January 2000 and March 2026 that evaluate BCI for cognitive function in patients with VCI. **Results:** A total of 30 studies were included in the review. The participants comprised 696 stroke patients, 71 patients with early cerebral microangiopathy and 128 patients with VCI and no dementia. In patients with VCI, BCI interventions combined with other technologies (e.g., exoskeleton, virtual reality, functional electrical stimulation, acupuncture, or game-based cognitive training) appeared more effective for cognitive rehabilitation than BCI alone. Attention was the most consistently improved domain among the studies reviewed. Global cognitive function also improved in many studies, though not uniformly. Memory, executive function, and language outcomes varied depending on factors such as intervention protocols, training duration, and assessment tools. **Conclusions:** BCI is a promising tool for cognitive assessment and rehabilitation in patients with VCI. However, substantial heterogeneity across studies limits the conclusions. Future large-scale, well-designed randomized controlled trials with standardized outcome measures are needed to validate the efficacy of BCI technology and to explore its underlying mechanisms.

## 1. Introduction

Vascular cognitive impairment (VCI) encompasses a broad spectrum of syndromes due to cerebrovascular lesions and their associated risk factors, ranging from mild cognitive impairment (MCI) to overt dementia [1]. VCI is commonly associated with stroke, cerebral small vessel disease, and other cerebrovascular conditions. Among these, stroke is an extremely prevalent vascular disease and remains the fifth leading cause of death worldwide [2]. Notably, within the first year after stroke, approximately 40% of patients develop cognitive impairment that does not meet the diagnostic criteria for dementia [3]. An even greater proportion experience cognitive impairment shortly after stroke, with up to 30% progressing to dementia within 5 years [4]. This trajectory of cognitive decline profoundly impairs critical cognitive domains, including attention, memory, executive function, and visuospatial abilities, thereby substantially reducing the quality of life for both patients and their families.

Currently, there are few targeted and effective treatments for VCI, and most patients rely on traditional pharmacological interventions, such as cholinesterase inhibitors (e.g., donepezil, galantamine, and rivastigmine) and low-affinity antagonist to N-Methyl-D-aspartate type receptors antagonists (e.g., memantine) [5,6], and other drugs that may confer modest cognitive benefits. However, these medications are often associated with side effects, including nausea, vomiting, and diarrhea, and more seriously, increased risk of adverse events such as stroke, pneumonia, and myocardial infarction [6,7,8]. Encouragingly, early identification of cognitive impairment and timely, intensive interventions have been associated with a reduced risk of cognitive impairment [9]. Against this background, brain–computer interface (BCI) technology, as an emerging therapeutic tool, has gradually shown great promise for application in cognitive recovery.

BCI technology was first proposed by Vidal in 1976, who defined the concept and developed a system to convert electroencephalography (EEG) into computer-controlled signals, thereby laying the foundation for subsequent BCI research [10]. Over several decades, BCI technology has evolved into a broad class of devices and techniques that establish direct communication pathways between the brain and external devices, enabling interaction with the external environment without reliance on the peripheral nervous system [11]. Specifically, BCI systems operate by acquiring neural electrical activity from different brain regions, decoding these electrophysiological signals and converting them into interpretable commands that allow computers or external devices to respond directly to brain activity [12].

**Figure 1:**
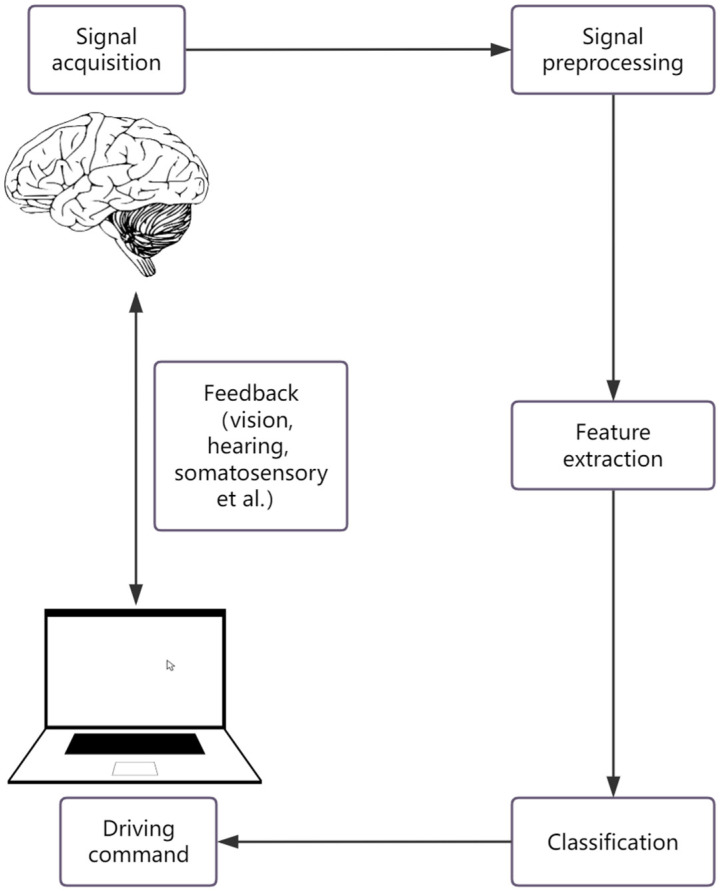
Basic framework of a BCI system.

A typical BCI system consists of five core components: signal acquisition, signal preprocessing, feature extraction, signal classification, and output control with user feedback [13] (Figure 1). Signal acquisition represents the initial and critical step, and may be achieved using either invasive or non-invasive methods. Signal preprocessing, feature extraction, and classification form the core of the signal processing module, which extracts meaningful information from noisy EEG data. These processed signals are then converted into control commands to drive external devices or provide real-time feedback to users, facilitating adaptive modulation of neural activity. Depending on the specific application, each component of a BCI system can be optimized to accommodate the unique needs of patients or clinical scenarios.

Neurofeedback training (NFT), a specific application of BCI technology, is a type of biofeedback in which individuals receive real-time representations of their neural activity through visual, auditory, or other sensory modalities. Based on this feedback, participants consciously modulate their brain activity to promote self-regulation of targeted neural substrates implicated in specific behavioral or pathological conditions [14,15]. In patients with VCI, abnormal neural activity patterns are commonly observed. Accordingly, NFT aims to modulate the EEG activity within specific frequency bands to enhance arousal levels and improve performance across multiple cognitive domains [16].

The brain has the ability to recover and reorganize itself from structure to function through learning and stimulation, which is called neuroplasticity [17]. The cognitive level of most patients improves somewhat in the first 12 months after stroke compared with the acute phase due to spontaneous recovery mediated by central nervous system plasticity [18,19,20]. However, these changes are generally insufficient to produce significant functional recovery and are still far from normal. Effective rehabilitation training can promote dynamic repair and reorganization within the nervous system. In fact, much of the research on various post-stroke recovery methods is related to the promotion of neuroplastic changes that reestablish or strengthen functional connectivity in damaged brain regions [21]. Similarly, cognitive improvements in patients with VCI are closely linked to the recovery of both brain structure and function.

A growing body of evidence has demonstrated the application of BCI in cognitive functions. Numerous clinical studies have explored the potential of BCI to enhance cognition in healthy older adults, as well as its role in cognitive rehabilitation across various conditions, including attention-deficit/hyperactivity disorder [22,23,24], stroke [25,26], and traumatic brain injury [27,28]. VCI is characterized by distinct pathophysiological mechanisms primarily related to cerebrovascular pathology, such as white matter hyperintensities, lacunar infarctions, and chronic cerebral hypoperfusion, which disrupt large-scale neural networks underlying cognitive processes [29]. These disruptions at the neural network level provide the theoretical basis for BCI applications, as BCIs precisely modulate distributed neural activity. BCI applications in VCI have primarily targeted two domains: cognitive assessment and cognitive rehabilitation. Several previous systematic reviews and meta-analyses have examined the use of BCI or neurofeedback (NF) interventions for cognitive or motor rehabilitation. Renton et al. conducted a systematic review of NF as a cognitive rehabilitation therapy following stroke, concluding that although NF was associated with improvements in cognitive deficits, the limited study quality and heterogeneity of protocols restricted the generalizability of the findings [30]. More recently, Vilou et al. reviewed EEG-NF for cognitive deficits across several neurological conditions, including stroke, traumatic brain injury, dementia and multiple sclerosis, noting the heterogeneity of protocols and the need for further high-quality trials [31]. In addition, a review focusing on NFT for MCI and Alzheimer’s disease reported generally positive effects on memory and attention [32]. However, these prior reviews predominantly focused on stroke populations or mixed patient cohorts, and none specifically addressed the full spectrum of VCI, including post-stroke cognitive impairment, subcortical ischemic vascular cognitive impairment, multi-infarct cognitive impairment and mixed cognitive impairment. These subtypes may differ in responsiveness to BCI-based interventions. It remains unclear whether findings derived from stroke populations can be safely and effectively applied to patients with other VCI subtypes. In addition, existing reviews have not systematically synthesized domain-specific cognitive outcomes, hindering the design of targeted rehabilitation strategies. Many current reviews have adopted either a technology-centered perspective, focusing on BCI paradigms such as motor imagery or P300-based systems, or a perspective that mainly emphasizes motor rehabilitation. Consequently, a systematic review that specifically examines BCI applications across the full spectrum of VCI is essential to identify current evidence, highlight knowledge gaps, and guide future mechanistic and clinical research. This review focuses on the clinical application of EEG-based BCI technologies in VCI, describing in detail the specific training tasks and outcomes across four major cognitive domains: attention, memory, executive function, language, and other cognitive processes.

## 2. Methods

### 2.1. Strategy Search

**Figure 2:**
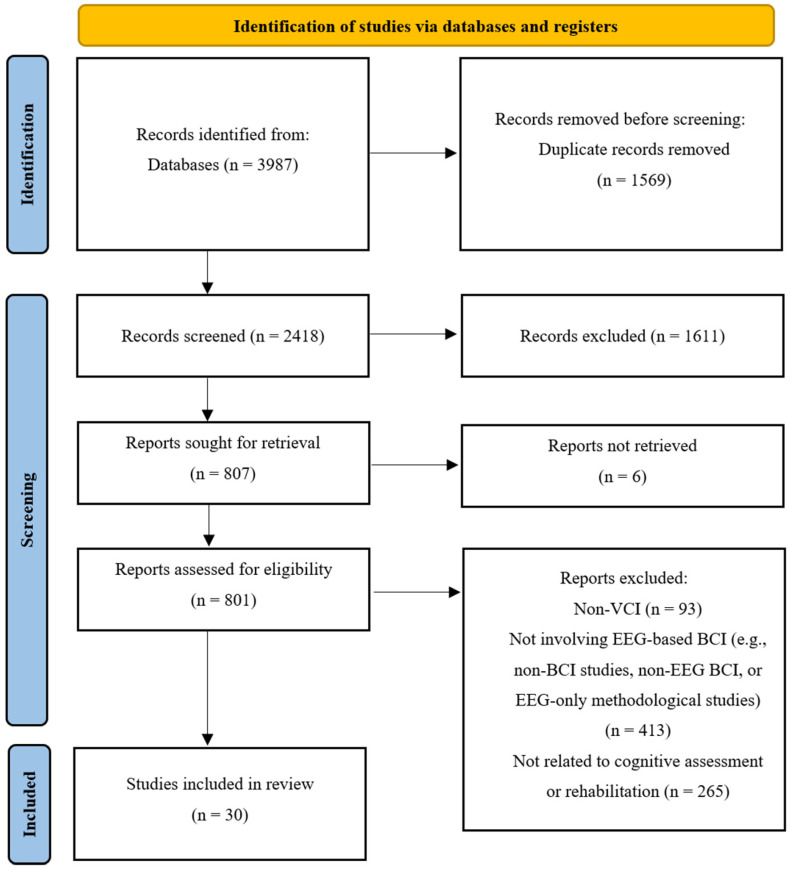
PRISMA flow diagram of the study selection process. Databases searched included PubMed, Medline and Web of Science, Embase, and CENTRAL.

To identify clinical research articles on the application of BCI in patients with VCI, a comprehensive literature search was performed in Medline, PubMed, Web of Science, Embase, and the Cochrane Central Register of Controlled Trials (CENTRAL). The systematic review was performed in accordance with the Preferred Reporting Items for Systematic Reviews and Meta-Analyses (PRISMA) 2020 guidelines [33] (the completed PRISMA 2020 checklist is provided in the Supplementary Materials). This systematic review was not prospectively registered. Nevertheless, the review was performed in strict accordance with the PRISMA 2020 checklist, ensuring a transparent, reproducible, and methodologically robust process. The search aimed to identify clinical and experimental studies investigating EEG-based BCI applications for cognitive assessment and rehabilitation in VCI. The primary cognitive domains of interest reflected the core or commonly affected deficits in VCI, including executive function, information processing speed, attention, and memory. Given the rapid development of BCI technology over recent decades, the search covered studies published from January 2000 to March 2026 to ensure that the most recent and relevant studies were captured at the time of analysis. The search strategy included combinations of the following core Medical Subject Headings, Emtree terms and keywords: (“brain–computer interface” OR “neural interface” OR “brain machine interface” OR BCI OR BMI OR electroencephalography OR EEG OR neurofeedback OR NF) AND (“vascular cognitive impairment” OR “vascular cognitive disorder” OR VCI OR stroke OR “post-stroke” OR poststroke OR PSCI OR “cerebral small vessel disease” OR “small vessel disease” OR CSVD OR “cerebral infarction” OR “cerebral hemorrhage” OR “cerebrovascular accident” OR “cerebrovascular disease” OR “brain ischemia” OR “vascular dementia”) AND (cogniti* OR “mild cognitive impairment” OR MCI OR memory OR “executive function” OR attention OR “processing speed”). The complete search strings for each database are provided in the Supplementary Materials. We also manually screened the reference lists of all included studies and relevant reviews to identify any additional eligible studies. The full search strategy is illustrated in Figure 2.

### 2.2. Eligibility Criteria

The inclusion criteria for eligible studies were as follows: (1) studies published between January 2000 and March 2026; (2) studies involving human participants diagnosed with VCI (defined according to internationally recognized guidelines such as the Vascular Impairment of Cognition Classification Consensus Study criteria, or, if diagnostic criteria were not reported, as patients with impairment in one or more cognitive domains, evidence of cerebrovascular disease on neuroimaging, and cerebrovascular disease as the primary contributor to cognitive impairment); (3) studies applying EEG-based BCI for cognitive assessment or rehabilitation; (4) studies reporting at least one cognitive outcome related to domains commonly affected in VCI; and (5) studies with eligible designs, including interventional studies (such as randomized controlled trials, non-randomized controlled trials, single-arm pre–post intervention studies, and pilot or feasibility studies) and observational studies (such as cross-sectional and case–control designs). Case reports were also included if they provided relevant information on cognitive outcomes related to BCI. The exclusion criteria were: (1) animal studies, in vitro studies, computational or simulation-only studies without human participants; (2) studies focusing on populations without vascular cognitive impairment; (3) studies involving populations with primary neurodegenerative disorders (e.g., Alzheimer’s disease or Parkinson’s disease) as the main etiology of cognitive impairment, unless VCI was explicitly confirmed as a primary or coexisting diagnosis; (4) studies not assessing or reporting cognitive outcomes; and (5) review articles, meta-analyses, editorials, conference abstracts without sufficient methodological detail, and protocol papers.

No restrictions were applied regarding patient age, sex, educational level, or the cognitive assessment methods employed. Additionally, given the diversity of clinical research designs, studies were included regardless of whether a control group was present.

Two reviewers (J.H. and T.M.) independently screened titles and abstracts to determine eligibility. Articles deemed potentially relevant were retrieved for full-text assessment, which was also conducted independently by the same reviewers. Any discrepancies between reviewers were resolved by discussion or consultation with a third reviewer (L.G.). The first search was performed on 24 December 2025, and an updated search was conducted on 16 April 2026.

### 2.3. Risk of Bias Assessment

Risk of bias was assessed using the Cochrane Risk of Bias 2 (RoB 2) tool (2019 version) for randomized controlled trials (RCTs) [34], and the Joanna Briggs Institute (JBI) critical appraisal tools for non-randomized studies [35,36]. For RCTs, the following six domains were evaluated: (1) bias arising from the randomization process, (2) bias due to deviations from intended interventions, (3) bias due to missing outcome data, (4) bias in measurement of the outcome, (5) bias in the selection of the reported result, and (6) other bias (e.g., technical specifications, intervention protocols, parameter configuration), which we added as an additional domain. For non-randomized and cross-sectional studies, the corresponding JBI design-specific checklists were applied in accordance with the study design. One reviewer performed the assessment for each study, and a second reviewer verified the results. Disagreements were resolved through discussion or, when necessary, by consulting with a third reviewer.

## 3. Results

### 3.1. Study Selection Process

A total of 3987 records were retrieved from the five databases. After removing 1569 duplicate records, 2418 records remained for initial screening. During the title and abstract screening stage, 1611 records were excluded for not meeting the inclusion criteria. Additional six records were excluded because they referred to ongoing trials without published results or because the full texts could not be obtained despite extensive efforts. The remaining 801 studies underwent full-text review, of which 771 were excluded. The main reasons for exclusion were as follows: studies involving populations without VCI or without explicit reporting of cognitive impairment (*n* = 93), studies not involving BCI (*n* = 385); studies not using EEG-based BCI (e.g., those based on cerebral blood flow signals, functional magnetic resonance imaging (fMRI), functional near-infrared spectroscopy, or electromyography) (*n* = 28); and studies not involving cognitive assessment or rehabilitation, or not reporting any cognitive outcome measures (*n* = 265). Finally, 30 studies met all inclusion criteria and were included in the review.

### 3.2. Patient Profile

A total of 30 studies involving 895 participants were included in this review. Among them, 696 participants were patients with stroke, 71 had early cerebral microangiopathy, and 128 had vascular cognitive impairment without dementia. Regarding study design, 15 studies were RCTs, eight were single-arm pre–post studies, two were non-randomized controlled trials, two were case reports, and three were cross-sectional studies. The sample sizes varied substantially across studies. Among the RCTs, the largest study included 100 participants, whereas the smallest enrolled only 10 participants. The sample size in the intervention groups was generally modest, typically ranging from 15 to 30 participants. Training duration typically consisted of 8–20 sessions within 2–12 weeks. Most studies were published in English (*n* = 28), with one study published in Chinese and one in Russian.

Due to considerable heterogeneity in intervention protocols, cognitive assessment methods, and outcome measures across studies, a quantitative synthesis was not considered appropriate. Therefore, the findings were analyzed and summarized using a descriptive and narrative approach, focusing on intervention strategies and changes in cognitive outcomes. The characteristics of the included studies were systematically summarized in Table 1, including authors, publication year, sample characteristics, intervention methods, intervention duration, primary outcomes, and the role of BCI.

### 3.3. Risk of Bias Assessment Results

**Figure 3:**
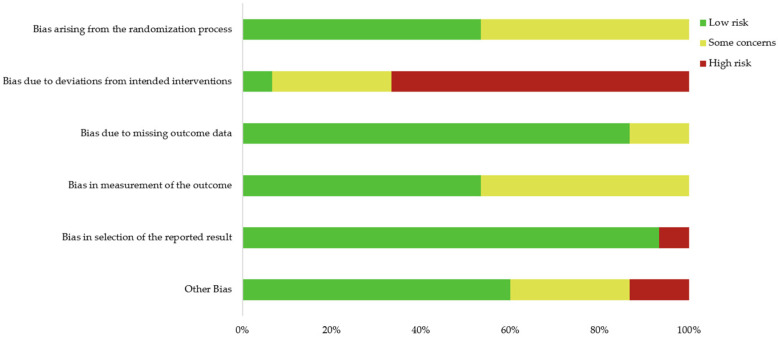
Risk of bias assessment (randomized controlled trials).

The quality of the included studies was evaluated using the RoB 2 and JBI critical appraisal tools. In the 15 RCTs, most studies showed low risk or some concerns across the evaluated domains, while a smaller proportion were judged as high risk (Figure 3). Eight studies were considered at low risk of bias arising from the randomization process. Seven had some concerns due to insufficient reporting of random sequence generation and allocation concealment procedures. Regarding bias due to deviations from intended interventions, only one double-blind placebo-controlled trial was at low risk. Four single-blind studies had some concerns, mainly because blinding of participants and personnel was difficult to implement in BCI, NFT, or physical rehabilitation interventions. The remaining 10 studies were at high risk owing to the absence of blinding. In terms of missing outcome data, 13 studies were at low risk. One study had some concerns (approximately 20% attrition without sensitivity analysis), and one study was judged at high risk due to substantial loss to follow-up. Concerning bias in measurement of the outcome, eight studies were at low risk, while seven had some concerns due to unclear blinding of outcome assessors or reliance on subjective outcome measures. With respect to selective reporting, 14 studies were at low risk, only one study was at high risk because it changed its primary outcome after trial registration. In addition, studies involving BCI, NFT, or virtual reality (VR) exhibited other potential biases, four studies had some concerns due to incomplete reporting of technical specifications, and two studies were at high risk because they provided no information on key methodological parameters, such as functional electrical stimulation (FES) settings, electrode placement, or algorithmic implementation. Although these aspects are not formally included within the RoB 2 domains, they were additionally considered in our assessment as they may influence intervention reproducibility and interpretability.

**Figure 4:**
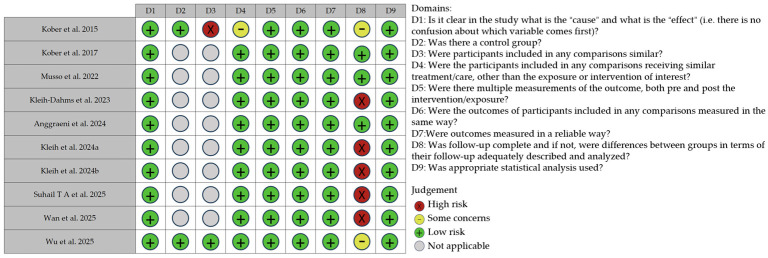
Risk of bias assessment (non-randomized controlled trials and pre–post studies) [43,44,50,53,55,56,57,60,61,63].

Among the 10 non-randomized studies, three were rated as having low risk of bias. One study was judged to be at high risk of bias due to substantial baseline imbalance between groups, lack of reporting on whether the intervention and control groups received comparable co-interventions, and inadequate handling of missing data. Another study was also rated as having some concerns due to inadequate handling of missing data. Five studies were considered at high risk of bias due to relatively high attrition rates (>20%) that were not adequately addressed (Figure 4).

For the two case reports, both were rated as having some concerns due to the lack of reporting on adverse events. All three cross-sectional studies were assessed as having a low risk of bias.

### 3.4. BCI-Based Cognitive Assessment

The application of BCI technology to develop new neuropsychological assessment tools can provide a more objective, accurate, and engaging approach to evaluating cognitive function in patients with VCI. Such BCI-based assessments typically quantify cognitive performance through participants’ execution of predefined tasks. Among the included studies, three cross-sectional studies explored the application of BCI-based cognitive assessment approaches in patients with stroke [38,39,49]. These studies incorporated steady-state visual evoked potential (SSVEP), VR, and task-induced paradigms to enable a more objective evaluation of cognitive function. Consistent with clinical expectations, stroke patients generally performed worse than healthy controls on these BCI-based assessment systems. Across three studies, cognitive indices derived from BCI technology showed moderate-to-strong correlations with traditional neuropsychological tests, with reported correlation coefficients ranging from approximately 0.60 to 0.80. Notably, three stroke patients with severe motor impairments, who were unable to complete the written Symbol Digit Modalities Test (SDMT), achieved accuracy rates exceeding 70% using the BCI-SDMT, demonstrating that BCI-based assessment can bypass motor output limitations.

### 3.5. BCI-Based Cognitive Rehabilitation

The cognitive domains commonly affected in VCI include memory, attention, executive function and language [65,66]. In patients with VCI, memory impairment is a frequent clinical feature and is often reflected in poor performance on neuropsychological measures of immediate and delayed recall. These deficits may also co-occur with disturbances in other cognitive domains, such as attention and executive control, which further compromise memory performance by reducing the efficiency of memory encoding strategies and the ability to retrieve information [67]. Attention impairments in stroke patients commonly manifest as deficits in sustained attention, selective attention, and attentional shifting. Affected individuals often experience difficulty maintaining concentration over extended periods and are highly vulnerable to distractions, leading to impaired task focus [68]. Attention is a critical prerequisite for motor recovery and the acquisition of new skills, making it essential for cognitive rehabilitation in patients with VCI. Notably, deficits in attention also have an impact on other higher-order cognitive functions, such as memory, executive function and language [69,70]. Executive function refers to a set of higher-level cognitive processes, including attention regulation, planning, problem solving, multitasking, and behavioral control, all of which are indispensable in performing novel or intricate tasks [71]. In VCI, executive dysfunction is considered a hallmark cognitive feature and often precedes prominent memory deficits [72,73]. Patients with VCI also frequently present with language dysfunction, typically characterized by reduced verbal fluency, impaired word retrieval, naming difficulties, and deficits in semantic processing. Collectively, these cognitive impairments may adversely affect patients’ social interaction, functional independence, and overall quality of life in daily activities.

The majority of included studies (*n* = 28) evaluated various BCI or NFT paradigms as cognitive rehabilitation interventions in VCI. The included populations comprised patients with stroke, as well as individuals described as having early cerebral microangiopathy and vascular cognitive impairment without dementia.

#### 3.5.1. Attention

Attention appears to be the most frequently reported cognitive domain among the included studies. Nine studies primarily evaluated the effects of BCI technology on attention, including six RCTs [40,41,47,52,54,62] and three pre–post studies [53,57,60]. Intervention duration typically consisted of 20–30 min each session over 2–4 weeks. Two studies adopted more intensive training schedules (up to 30 sessions over 6 weeks), while one study employed a brief intervention of three sessions and still reported improvements.

Among the RCTs, intervention protocols were heterogeneous. Several studies used BCI-controlled pedaling or FES with real-time feedback based on EEG metrics [41,47,54,62]. Others employed P300-based BCI speller tasks [52], or NFT targeting sensorimotor rhythm, beta band activity or sample entropy [40]. Attention was assessed using a range of measures, such as the Montreal Cognitive Assessment (MoCA), attention subscale, the SDMT, the Trail Making Test (TMT), the Attention Network Test, the Digit Vigilance Test, the Schulte Grid Test, and EEG-derived indices (beta/alpha ratio, attention concentration quotient). All six RCTs reported statistically significant improvements in at least one attention measure in the intervention group compared with baseline or a control group. However, the pattern and consistency of these improvements varied across studies. For example, Fateeva et al. found a large increase in MoCA attention scores (from 2.3 to 5.2 points), while the control group showed a decline [52]. In a small-sample RCT (*n* = 5 per group), significant increases were found only in frontopolar EEG-derived attention indices in the BCI-FES group [41]. In addition, the presence or absence of real-time visual feedback has a significant impact on attention enhancement [62]. In a MI-VR-based dual-task paradigm, improvements in SDMT performance were accompanied by gains in balance, and the effect was maintained at 4-week follow-up [62]. However, the current evidence remains limited. One study reported improvements in SDMT performance without corresponding changes in TMT-A, suggesting that gains in attention are not consistently observed across different cognitive measures [47]. Most studies lack long-term follow-up after the end of training. Only one study with a 4-week follow-up suggests that the benefits are sustained [62]. Overall, the current limited but generally consistent evidence from RCTs suggests that BCI technology can improve attention function in patients with VCI. However, the selection of optimal protocols, the dose–response relationship of training, and long-term effects remain to be clarified through further research.

Three pre–post studies also examined the effects of BCI technology on attention in stroke patients. One study evaluated 20 sessions of slow cortical potential (SCP) NF in five chronic stroke patients with attention deficits and reported improvements in divided attention and attentional flexibility as measured by the Test Battery for Attention Performance (TAP). However, no significant changes were found in phasic alertness, working memory, or go/no-go performance, and the effects on daily life attention difficulties were not significant [53]. The second study delivered eight sessions of SCP NF to 16 chronic stroke patients. The authors reported a non-significant trend toward increased SCP negativity. Although some improvements were observed in divided attention and flexibility, the changes were not consistent across all measures. Notably, the study concluded that the results were inconclusive regarding the efficacy of SCP NF for post-stroke attention deficits [57]. Additionally, a short intervention consisting of three sessions of a BCI-based NF game led to increased EEG-derived attention indices in some patients, but not all participants, and the improvements were not compared with a control condition [60].

#### 3.5.2. Memory

Memory impairment, particularly verbal short-term and long-term memory, working memory, and visuospatial recall, is also a cognitive function significantly affected by VCI. Four studies evaluated the effects of BCI technology on memory, including one RCT [64], one non-RCT [43], and two pre–post studies [44,60]. All included participants were patients with stroke. The RCT implemented 20 sessions over 3 weeks, whereas the non-RCT delivered 10 sessions within 2–3 weeks. In contrast, one pre–post study employed a brief intervention consisting of only three sessions [60]. Memory outcomes were also assessed using a range of standardized neuropsychological instruments, including the California Verbal Learning Test, the Visual and Verbal Memory Test, the Wechsler Memory Scale, Rey’s Auditory Verbal Learning Test, the Rey–Osterrieth Complex Figure Test, and Digit Span measures.

Evidence from one small RCT suggested that NFT and brainwave entrainment may improve visual memory but not verbal memory in subacute stroke [64]. One non-RCT and one pre–post study provided lower-level evidence that SMR NFT may enhance verbal long-term memory and visuospatial short-term memory, whereas upper alpha NFT appeared to improve verbal working memory [43,44]. The pre–post study, which included only three sessions, reported potential memory gains without a control comparison. Overall, the available evidence suggests possible domain-specific effects of SMR and upper alpha NFT on memory subtypes [60]. However, the limited number of studies, small sample sizes, and lack of long-term follow-up limit definitive conclusions.

#### 3.5.3. Executive Function and Information Processing Speed

Three RCTs focused primarily on executive functions and information processing speed [42,58,64]. The patients included those with subacute stroke and early cerebral microangiopathy. The intervention protocols varied across studies. Two studies in stroke patients applied SMR NF over 24 sessions or alpha enhancement combined with theta suppression/brainwave entrainment (BWE) over 20 sessions, while a third study in early cerebral microangiopathy patients compared infra-low frequency NF with alpha NF and a placebo condition over 15 sessions. Outcome measures also differed and included dual-task paradigms (e.g., walking while performing serial subtraction), the Digit Symbol Substitution Test (DSST), TMT-A, the Stroop interference test, and phonemic fluency tasks.

Across the three RCTs, improvements were reported in at least one measure of executive function or processing speed following active intervention. In patients with subacute stroke, SMR NF was associated with improved dual-task performance, reflected by reduced errors during simultaneous motor and cognitive tasks, along with concurrent improvements in gait parameters [42]. Another study reported that both NF and BWE improved processing speed, as measured by the DSST, and performance on a delayed complex figure recall task, which reflects executive-memory integration [64]. In patients with early cerebral microangiopathy, infra-low frequency biofeedback was associated with improvements in processing speed and executive control, as indicated by reduced completion time on the TMT-A and improved Stroop interference performance. In contrast, alpha-frequency NF did not result in significant changes in executive outcomes [58].

#### 3.5.4. Language

Language deficits, particularly aphasia, after stroke profoundly affect communication, social participation, and quality of life. Among the three included studies on improvements in language function, the effects of BCI technology varied significantly [45,50,55]. Two of these studies employed the P300 paradigm: one involved auditory P300-based language training [50], and the other involved visual P300-based speller training [55]. The case report utilized alpha-band NFT [45].

Both P300-based BCI studies used the Aachen Aphasia Test (AAT) as the primary language assessment, which includes subtests for spontaneous speech, token test, repetition, written language, naming, and comprehension. In these two studies, BCI-based language training was associated with improvements across multiple linguistic domains, including naming, repetition, written language, and spontaneous speech. More pronounced and consistent improvements were observed in the auditory P300 paradigm, with gains extending across all AAT subtests and maintained at 3-month follow-up. Notably, five out of ten patients were classified as non-aphasic after training according to AAT criteria. EEG analysis revealed an increase in P300 amplitude and a shortened latency, while resting-state fMRI indicated enhanced functional connectivity in the left language network and a rebalancing between the default mode network and the language network [50]. In contrast, the visual P300 paradigm yielded more modest and less consistent changes, with improvements limited to repetition, written language and spontaneous language. Only three of the seven participants showed a reduction in aphasia severity [55]. Nan et al. reported that two patients with chronic stroke underwent 15 sessions of alpha-band NFT. The results showed improvements in word naming, sentence completion, and verbal fluency. However, no statistical tests were conducted, and no control group was established [45]. Overall, the auditory P300-based BCI demonstrated more robust, broader, and sustained language improvements, whereas the visual P300 paradigm showed limited effects, and alpha NF provided only preliminary observational evidence. These findings suggest that the potential of BCI technology for language rehabilitation may be closely linked to the choice of paradigm.

#### 3.5.5. Multidimensional Cognitive Rehabilitation

Beyond focusing on individual cognitive domains, many studies adopted multidimensional approaches to evaluate recovery across multiple cognitive domains simultaneously. There were 10 studies that reported improvements across more than two cognitive domains or on global cognitive scales. The investigated populations were predominantly stroke patients, whereas fewer studies focused on patients with early or non-demented cerebral microangiopathy. Study types included seven RCTs [25,26,46,48,51,59], two pre–post studies [56,61], one non-RCT [63], and one case report [37]. Across these studies, the MoCA was the most commonly used measure, while some studies also employ neuropsychological tests targeting specific cognitive domains. The most common training time was 8–20 sessions, with session durations typically ranging from 20 to 40 min. There was no clear dose–response relationship observed across studies, but training protocols with at least 10 sessions tended to yield more consistent improvements than those with fewer sessions.

Eight studies reported global cognitive outcomes using the MoCA as a primary or secondary measure [25,26,46,48,56,59,61,63]. Overall, BCI technology was consistently associated with increases in MoCA scores, with mean improvements ranging from approximately 1.6 to 5.5 points. In RCTs, both using BCI technology alone and BCI combined with other training methods have shown beneficial effects. Paradigms that combine BCI with exoskeleton training [48], FES [26], acupuncture [59] or additional cognitive/sensorimotor stimulation [46] tend to yield greater improvement compared with control conditions involving BCI alone or no BCI intervention. Studies comparing different paradigms indicated that both P300-based and mu-rhythm-based BCI were associated with improved global cognition, although P300-based systems appeared to be effective even in patients with lower baseline cognitive performance, whereas mu-rhythm-based approaches may require relatively preserved cognitive function [25]. Pre–post studies also supported these findings, showing improvements in MoCA after NFT or BCI combined with virtual reality [56,61], often accompanied by improvements in attention and processing speed. However, longitudinal evidence remains limited. One study with extended follow-up reported results at 4.5 years in only 24 of the original 100 patients. In this study, MoCA scores peaked at approximately 4 months after intervention but returned to near baseline levels by 4.5 years, suggesting that age-related cognitive decline may offset the initial intervention benefits over the long term [26].

In addition to global cognitive outcomes, several studies assessed multidomain cognitive changes using alternative neuropsychological measures. An RCT employing the Loewenstein Occupational Therapy Cognitive Assessment demonstrated significant improvements across multiple domains, including attention, memory, visuospatial function, and executive abilities, following BCI-controlled robotic training compared with a sham condition [51]. Attention and processing speed emerged as the most consistently responsive domains, followed by executive functions such as cognitive flexibility and dual-task performance [51,61]. Memory-related improvements, particularly verbal and working memory, were also reported but showed greater variability across studies. Multidomain gains were frequently accompanied by functional improvements in motor performance, activities of daily living, or affective symptoms, suggesting that the processes of cognitive, motor, and emotional recovery partially overlap [37,59,63].

### 3.6. Adverse Events

Across the 30 included studies, no serious adverse events related to BCI-based technologies were reported. However, minor and transient adverse effects were noted in several studies. In a cross-sectional study using VR-based cognitive assessment, Kang et al. [38] reported simulator sickness in some stroke patients (nausea: 9.6%, oculomotor: 41.9%, and disorientation: 25.8%), with no significant differences compared to healthy controls. Approximately 35% of stroke patients reported difficulty operating the joystick interface, highlighting the need for more intuitive interface designs. Similarly, Ku et al. [39] noted that some patients experienced discomfort associated with the head-mounted display, although no serious complications were reported.

Fatigue and frustration were also reported, particularly in studies requiring long or intensive training sessions. In the SCP neurofeedback study by Kleih et al., participants reported fatigue and mental exhaustion after prolonged sessions. Some participants also expressed frustration due to difficulty in achieving BCI control or slow progress [55]. Several studies explicitly stated no adverse events or equipment issues, including Wan et al. [62], Musso et al. [50] and Chen et al. [26].

In summary, BCI-based technologies appear to be safe, with no serious or persistent adverse effects. The most frequently reported issues were transient simulator sickness in VR-based paradigms and fatigue/frustration in intensive training protocols. However, most included studies did not mention whether adverse events were monitored or occurred. The above summary reflects only those studies that provided explicit information. This lack of systematic adverse event reporting limits the assessment of safety for BCI-based technologies in VCI.

## 4. Discussion

This systematic review synthesized evidence from 30 studies involving 895 participants with VCI, indicating that BCI technologies are feasible and potentially beneficial for both cognitive assessment and rehabilitation. The included studies encompassed a range of BCI paradigms, including oscillatory activity-based NFT approaches aimed at modulating specific EEG rhythms to regulate cortical excitability and cognitive processing. P300-based, SSVEP-based, SMR-based and SCP-based paradigms targeted voluntary regulation of brain signals related to cognitive control, attention, and language processing. In addition, closed-loop BCI systems integrated with external feedback modalities or task environments were employed, such as virtual reality platforms, motor imagery-based pedal systems, FES, robotic exoskeletons, and game-based cognitive training interfaces. Most studies used small to moderate sample sizes, reflecting the emerging nature of this field in vascular cognitive impairment populations.

In the context of cognitive assessment, three studies indicated that BCI-based cognitive assessment systems may be feasible for evaluating cognitive function in patients with VCI, particularly in those with motor impairments. Potential advantages such as real-time interaction, immediate feedback, and accurate performance recording allow for a more dynamic and ecologically valid assessment process. However, all three studies were preliminary and none provided normative data or diagnostic accuracy metrics. It remains unclear to what extent these differences are attributable to cognitive impairment rather than task-specific or interface-related factors. In addition, the very small sample sizes limit the generalizability of the findings. Given the heterogeneity in study designs, assessment protocols, and the relatively limited evidence base, these approaches are more likely to serve as complementary tools rather than replacements for traditional paper-based tools in the near future. Further well-designed studies are needed to establish their validity, reliability, and clinical utility across diverse patient populations.

In the context of cognitive rehabilitation, 27 studies evaluated BCI or NF interventions for cognitive rehabilitation in patients with VCI. Among the included studies, most reported positive findings in at least one cognitive domain, including attention, memory, executive functions and processing speed, language, or global cognition, and the observed cognitive benefits may depend on the specific paradigm. However, the considerable methodological heterogeneity, small sample sizes, lack of rigorous blinding, and limited use of sham controls in most studies indicate that these findings should be interpreted as preliminary rather than definitive and conclusive. P300-based BCI [46,52], BCI-controlled pedaling task [47,62] and BCI combined with FES [41,54] showed relatively consistent improvements in attention across several small RCTs, even in patients with low baseline MoCA scores. In contrast, SCP NF yielded inconsistent results, as one study reported partial improvements in divided attention and flexibility [53], whereas another found no significant changes in most attention measures [57]. Consequently, based on the available evidence, the efficacy of SCP NF for post-stroke attention deficits remains unsubstantiated. SMR NF was more consistently associated with improvements in memory, particularly verbal long-term and visuospatial short-term memory, whereas upper alpha training appeared more relevant for working memory [43,44]. Infra-low frequency biofeedback demonstrated enhancements in processing speed and inhibitory control in a double-blind, placebo-controlled trial. While this protocol represents the most rigorously tested approach in this domain, it has not yet been replicated in other samples or settings. The auditory P300 paradigm appeared more promising than visual P300 speller training, with broader and more sustained language gains reported in chronic aphasia. Following training with the auditory P300 paradigm, neuroimaging changes were observed in the brain, characterized by increased functional connectivity within the left language network and a rebalancing between the language network and the default mode network. In contrast, the visual P300 paradigm may not sufficiently engage phonological and semantic processing [50,55]. Studies reporting improvements in multiple cognitive domains or global cognition employed diverse BCI paradigms, and the heterogeneity of these interventions precludes direct comparisons of efficacy across paradigms. Notably, P300-based BCI appeared effective even in patients with very low baseline MoCA (20 points or lower), whereas μ rhythm BCI required a baseline MoCA score of 22 or higher to achieve outcomes superior to standard training [25,48]. This suggests that patient selection may be as important as paradigm selection. Multimodal approaches combining BCI with exoskeleton, VR, acupuncture, FES or cognitive training frequently produced broader improvements than BCI alone [26,38,43,51,58,59]. However, because these protocols also involved higher intensity or additional active components, the unique contribution of multimodality remains unclear. No single BCI paradigm can be identified as definitively more effective for global cognition based on current evidence. Overall, few studies incorporated mechanistic biomarkers to explore neuroplastic changes, and the current evidence remains preliminary and does not support definitive conclusions regarding the superiority of any specific BCI technology. There is a need for large-scale, sham-controlled and high-quality RCTs with standardized outcome measures and extended follow-up to establish which paradigms can consistently produce clinically meaningful cognitive benefits in patients with VCI. Future reviews that include more technology-oriented databases may help better identify differential efficacy across BCI paradigms by capturing a broader range of technical and clinical studies.

It is important to note that most participants in the included studies were stroke patients (696/895, 77.77%), while other forms of VCI were relatively underrepresented. The current evidence primarily reflects the effects of BCI in patients with stroke. This imbalance may limit the generalizability of the findings to the broader VCI population. Different VCI subtypes are characterized by distinct patterns of brain injury, which may influence responsiveness to BCI interventions. For example, post-stroke lesions are typically focal and heterogeneous, whereas small vessel disease is associated with more diffuse white matter disruption and widespread network disconnection. These differences may potentially lead to different training responses or required intervention intensities. Patients with focal cortical lesions might benefit from frequency-specific protocols (e.g., SMR or upper-alpha training) that target local oscillatory activity, whereas those with diffuse white matter pathology may require global excitability regulation, such as infra-low frequency biofeedback, or extended training durations to achieve detectable cognitive gains. None of the included studies performed subgroup analyses based on VCI subtype, and most did not report separate results for different etiologies. Therefore, the positive effects observed in predominantly stroke samples should be interpreted cautiously when applied to other VCI subtypes.

The substantial heterogeneity among the included studies constrains the generalizability of the findings. Patient populations varied widely in etiology, lesion location, time since onset, and baseline cognitive severity, all of which are likely to influence both cognitive profiles and responsiveness to intervention. These factors highlight the need for more homogeneous study populations or for robust studies that include stratification or subgroup analyses. BCI paradigms also varied widely and each paradigm has different neural targets, feedback modalities, and training intensities. This diversity makes it impossible to identify a single optimal protocol, but it also suggests that BCI technology may be flexibly tailored to individual patient profiles. Outcome measures were not standardized. A wide variety of cognitive tests were used across studies, and many studies relied on a single global scale such as the MoCA rather than domain-specific neuropsychological batteries. Although the MoCA has shown greater sensitivity than the Mini-Mental State Examination in detecting cognitive impairment in patients with MCI, post-acute stroke cognitive dysfunction, it may still miss certain subtle changes in specific cognitive functions [74]. Therefore, future studies could combine global measures with domain-specific assessments to provide a more comprehensive assessment of cognitive outcomes. Overall, the substantial heterogeneity across studies precludes quantitative synthesis and further limits the comparability, generalizability, and reproducibility of individual findings. Accordingly, the interpretation of current evidence should be considered cautiously, and conclusions should be regarded as exploratory rather than confirmatory, with insufficient evidence to support strong clinical recommendations.

There are also several methodological limitations. A major limitation across the reviewed studies is the relatively small sample sizes, which restrict statistical power and limit the generalizability of findings. Many positive results stem from exploratory studies lacking a control group, which carry a high risk of bias. For instance, NFT interventions conducted by Kober et al. [44], Nan et al. [45], and Kleih-Dahms et al. [53] involved only a handful of participants, often fewer than five. While these studies provide important insights, small cohorts impede the ability to account for individual variability in response to BCIs, such as differences in baseline cognitive status, lesion characteristics, or psychological factors. Future research should aim to recruit larger and more diverse participant samples, ideally across multiple clinical centers, to more robustly validate both the efficacy and mechanisms of these interventions.

Another notable limitation lies in the insufficient exploration of long-term effects and the sustainability of cognitive improvements elicited by BCI interventions. Most research has focused on immediate post-training outcomes without conducting longitudinal follow-up, and only one study followed patients for more than one year and reported a decline in MoCA gains over 4.5 years. Long-term investigations are essential to determine whether repeated or maintenance sessions are necessary to consolidate cognitive gains and to identify optimal training durations and frequencies for enduring benefit.

Moreover, the mechanisms underlying the observed cognitive improvements remain poorly understood. Few studies have linked neurophysiological changes with improvements in cognitive performance. As a result, the relationship between brain activity modulation and behavioral gains remains largely indirect. The lack of integration between neuroimaging techniques and cognitive rehabilitation limits the understanding of the neural mechanisms of cognitive recovery. Investigating the biological mechanisms underpinning BCI-mediated cognitive rehabilitation could substantially enhance our understanding of post-stroke cognitive recovery. Future studies may benefit from adopting multimodal approaches that combine EEG, fMRI, and other neuroimaging modalities to provide a more detailed characterization of how BCIs influence neural networks and promote cognitive recovery.

The introduction of VR-based cognitive assessments, exemplified by Kang et al. [38] and Ku et al. [39], offers a novel approach for evaluating behavioral and executive functions in VCI patients who are difficult to assess with traditional paper-based tests. However, such systems may encounter technical and user interface challenges, particularly for elderly stroke patients or those with severe motor impairments. BCI-based technologies may also induce fatigue, discomfort, or simulator sickness, which may affect performance and limit clinical usability. Although some studies have demonstrated efficacy in cognitive assessment and rehabilitation, their clinical application may be limited by these transient adverse events and usability limitations. Moreover, most studies did not systematically report adverse events, which hampers a comprehensive safety evaluation. To address these limitations, future VR platforms should prioritize user-friendly designs and adaptive interfaces that accommodate varying levels of cognitive and physical ability. Incorporating shorter, modular tasks may further help minimize fatigue while maintaining diagnostic accuracy. Standardized monitoring and reporting of adverse events should be adopted in future studies to enhance transparency, comparability, and the evidence base for clinical implementation.

The risk of bias assessment revealed several methodological concerns across the included studies. The majority of RCTs lacked adequate blinding of participants and personnel, and few employed sham or placebo control conditions. The absence of blinding may introduce performance and detection biases, particularly when subjective outcome measures are used, and participants’ awareness of group allocation could further influence psychological factors, potentially affecting intervention outcomes. In addition, many studies did not provide detailed reporting of BCI parameters, which hinders reproducibility. High dropout rates raise concerns about the robustness and generalizability of the findings. Consequently, these methodological limitations may systematically inflate the observed positive effects.

Additionally, the high cost and the need for specialized equipment and substantial personnel time to set up and monitor the systems may make the BCI technology less accessible. For example, Kleih et al. reported that each training session required approximately 60–90 min from electrode preparation to completion [55]. In addition, most studies were conducted in supervised laboratory or clinical settings, which ensures safety and data quality but limits scalability. Home-based BCI technology has rarely been explored. One study suggested its feasibility in chronic stroke patients, but key implementation aspects such as remote monitoring and data quality control were not addressed, and no direct comparison with clinic-based training was performed [45]. Therefore, further exploration of home-based BCI devices is of great significance. They improve access to and adherence to training by reducing time and financial costs and alleviating the burden of traveling to and from medical facilities on patients, while also enhancing the sustainability and ecological validity of interventions through a more flexible, long-term home training environment. Furthermore, the development of simple, low-cost, portable BCI systems helps advance this technology from research into clinical practice.

## 5. Conclusions

This review of 30 studies suggests that BCI technology represents an emerging adjunctive tool for cognitive assessment and rehabilitation in patients with VCI, particularly in those with stroke. The efficacy of the broader spectrum of VCI remains to be established. However, the evidence remains limited and should be interpreted with caution. Small sample sizes, heterogeneous intervention protocols, inadequate blinding, and the absence of long-term follow-up constrain the strength and generalizability of the findings. Therefore, the application of BCI in clinical practice should be guided by individual patient characteristics and implemented within carefully monitored clinical or research contexts. Future well-designed, adequately powered RCTs with standardized outcome measures, appropriate control conditions, and extended follow-up are needed to further clarify efficacy, durability, and optimal intervention strategies.

Furthermore, personalized and multimodal BCI interventions may represent an important future direction. Selecting appropriate BCI paradigms based on patients’ cognitive characteristics, lesion types, and neurophysiological indicators and combining BCI with complementary approaches (e.g., VR, FES, exoskeleton, cognitive training) may enhance the efficacy of interventions and their ecological validity. However, the clinical benefits of these strategies still require further validation through high-quality research.

## Figures and Tables

**Table 1:** BCI in cognitive recovery of VCI patients.

Study	Samples	Tasks	Therapy Duration	Positive Outcomes?	Role
Bearden et al. [37] (2003)	1 stroke patients	NFT (theta reduction)	14 weeks, 2–3 d/w, 33 m/d	Yes (verbal recall, reading, visual tracking, and emotional stability have been improved)	Training and rehabilitation
Kang et al. [38] (2008)	20 stroke patients and 20 healthy controls	VR shopping simulation	3 stages, the duration is determined by patient performance	Yes	Assessment
Ku et al. [39] (2009)	20 stroke patients	VET-based shopping simulation	-	Yes	Assessment
Cho et al. [40] (2015)	27 stroke patients	NFT (mid-beta or SMR)	6 weeks, 5 d/w, 30 m/d	Yes (concentration and visual perception have been improved)	Training and rehabilitation
Chung et al. [41] (2015)	10 stroke patients	BCI-FES	5 sessions, 30 m/d	Yes (attention and brain activation have been more effectively stimulated)	Training and rehabilitation
Lee et al. [42] (2015)	20 stroke patients	NFT (SMR)	8 weeks, 3 d/w, 30 m/d	Yes (the dual-task performance and gait parameters have been improved)	Training and rehabilitation
Kober et al. [43] (2015)	24 stroke patients and 40 healthy controls	NFT (SMR or upper alpha)	10 sessions, 3–5 d/w, 45 m/d	Yes (visuo-spatial short-term memory and working memory have been improved)	Training and rehabilitation
Kober et al. [44] (2017)	2 stroke patients and 24 healthy controls	NFT (upper alpha)	10 sessions, 3–5 d/w, 45 m/d	Yes (memory function has been improved)	Training and rehabilitation
Nan et al. [45] (2019)	2 stroke patients	NFT (alpha enhancement)	15 sessions, 2 d/w, 60 m/d	Yes (language function has been improved)	Training and rehabilitation
Kotov et al. [46] (2020)	44 stroke patients	complex multimodal stimulation	8–10 sessions	Yes (memory, attention, visual land and constructive skills have been improved)	Training and rehabilitation
Yuan et al. [47] (2021)	30 stroke patients	BCI-controlled pedaling training system	24 sessions, 2 weeks, 6 d/w	Yes (attention has been improved)	Training and rehabilitation
Kotov et al. [48] (2022)	34 stroke patients	a. NFT (P300) b. BCI + MI-based exoskeleton training c. Computerized cognitive training	8–10 sessions, a. 15–30 m/t; b. 30 m/t; c. 15–30 m/t	Yes (global cognitive function and memory have been improved)	Training and rehabilitation
Chen et al. [49] (2022)	9 stroke patients and 89 healthy elderly	SSVEP-based BCI SDMT	10 min	Yes	Assessment
Musso et al. [50] (2022)	10 stroke patients and 20 healthy controls	P300-based BCI	30 h, 4 d/w	Yes (language has been improved)	Training and rehabilitation
Zhao et al. [51] (2022)	28 stroke patients	BCI-controlled robot training	4 weeks, 6 d/w, 60 m/d	Yes (global cognitive function has been improved)	Training and rehabilitation
Fateeva et al. [52] (2023)	30 stroke patients	P300-based BCI	10 days, no more than 60 m/d	Yes (global cognitive function, primarily attention have been improved)	Training and rehabilitation
Kleih-Dahms et al. [53] (2023)	5 stroke patients	NFT (SCP)	20 sessions, 60 m/d	Yes (attention has been improved)	Training and rehabilitation
Liu et al. [54] (2023)	60 stroke patients	MI-based BCI + FES	3 weeks, 5 d/w, 20 m/d	Yes (attention has been improved)	Training and rehabilitation
Kleih et al. [55] (2024)	7 stroke patients	P300-based BCI	12–20 sessions, 60–90 m/d	Yes (language has been improved)	Training and rehabilitation
Anggraeni et al. [56] (2024)	8 stroke patients	NFT (alpha or SMR)	2 weeks, 5 d/w, 30 m/d	Yes (global cognitive function has been improved)	Training and rehabilitation
Kleih et al. [57] (2024)	16 stroke patients	NFT (SCP)	8 sessions, 3–4 weeks	Yes (divided attention has been improved)	Training and rehabilitation
Chen et al. [26] (2025)	100 stroke patients	MI-based BCI + FES		Yes (global cognitive function has been improved)	Training and rehabilitation
Dobrynina et al. [58] (2025)	71 early cerebral microangiopathy patients and 2 healthy controls	NFT (infra-low waves or alpha waves)	15 sessions, 2–5 d/w, 30 m/d	Yes (executive function has been improved)	Training and rehabilitation
Lu et al. [59] (2025)	40 stroke patients	BCI + acupuncture therapy	8 weeks, 20 m/d	Yes (global cognitive function has been improved)	Training and rehabilitation
Suhail et al. [60] (2025)	6 stroke patients, 7 MCI patients and 2 healthy controls	NFT (sample entropy)	Over 3 sessions on consecutive 3 days	Yes (memory and attention abilities have been improved)	Training and rehabilitation
Wan et al. [61] (2025)	9 stroke patients	MI-VR-based pedaling training program	4 weeks, 5 d/w, 30 m/d	Yes (global cognitive function, attention and processing speed have been improved)	Training and rehabilitation
Wan et al. [62] (2025)	30 stroke patients	MI-VR-based dual-task modal BCI	4 weeks, 5 d/w, 20 m/d	Yes (attention has been improved)	Training and rehabilitation
Wu et al. [63] (2025)	128 VCI-ND	NFT (theta reduction, SMR, alpha and low beta enhancement)	12 weeks, 2 d/w, 30 m/d	Yes (global cognitive function has been improved)	Training and rehabilitation
Gupta et al. [64] (2026)	15 stroke patients	NFT (alpha and theta) or BWE	20 sessions over 3 weeks, 20 m/d	Yes (processing speed and memory have been improved)	Training and rehabilitation
Isakova et al. [25] (2026)	89 stroke patients	P300-based BCI or μ-rhythm-based BCI + NFT	8–10 sessions, 20–30 m/d	Yes (global cognitive function has been improved)	Training and rehabilitation

Abbreviations: w, week; d, day; m, minutes; t, time; NFT, neurofeedback training; VR, virtual reality; VET, virtual environment technology; BCI, brain–computer interface; SMR, sensorimotor rhythm; FES, functional electrical stimulation; MI, motor imagery; SSVEP, steady-state visual evoked potential; SDMT, symbol digit modalities test; SCP, slow cortical potentials; MCI, mild cognitive impairment; VCI-ND, vascular cognitive impairment-no dementia; BWE, brainwave entrainment.

## Data Availability

No new data were created or analyzed in this study. Data sharing is not applicable to this article.

## Supplementary Materials

The following supporting information can be downloaded at https://www.mdpi.com/article/10.3390/brainsci16060589/s1: File S1: PRISMA 2020 checklist, File S2: systematic review databases search strings.

## Abbreviations

The following abbreviations are used in this manuscript:

BCI	Brain–computer interface
VCI	Vascular cognitive impairment
MCI	Mild cognitive impairment
EEG	Electroencephalography
NFT	Neurofeedback training
NF	Neurofeedback
CENTRAL	Cochrane Central Register of Controlled Trials
PRISMA	Preferred reporting items for systematic reviews and meta-Analysis
RoB	Risk of Bias
RCT	Randomized controlled trial
JBI	Joanna Briggs Institute
fMRI	Functional magnetic resonance imaging
VR	Virtual reality
SSVEP	Steady-state visual evoked potential
VET	Virtual environment technology
SDMT	Symbol digit modalities test
SMR	Sensorimotor rhythm
MI	Motor imagery
SCP	Slow cortical potential
MoCA	Montreal Cognitive Assessment
FES	Functional electrical stimulation
TMT	Trail Making Test
BWE	Brainwave entrainment
DSST	Digit Symbol Substitution Test
AAT	Aachen Aphasia Test
