# Supplementary material for: Application of Brain–Computer Interface Technology in Vascular Cognitive Impairment: A Systematic Review
Source: Brain Sci. 2026 May 29;16(6):589. doi: 10.3390/brainsci16060589 (PMC13297284; doi:10.3390/brainsci16060589)
Supplement: Supplementary file 1 [file brainsci-16-00589-s001.zip › File S2. systematic review databases search strings.pdf]

PubMed

((((((((((brain-computer interfaces[MeSH Terms]) OR (brain-computer interface[Title/Abstract])) OR (BCI[Title/Abstract])) OR (brain-machine interface[Title/Abstract])) OR (brain-machine interfaces[Title/Abstract])) OR (BMI[Title/Abstract])) OR (neural interface[Title/Abstract])) OR (neurofeedback[Title/Abstract])) OR (electroencephalography[Title/Abstract])) OR (EEG[Title/Abstract])) AND ((((((((((((cognitive dysfunction[MeSH Terms]) OR (cogniti*[Title/Abstract])) OR (cognitive impairment[Title/Abstract])) OR (cognitive disorder[Title/Abstract])) OR (cognitive decline[Title/Abstract])) OR (cognitive function[Title/Abstract])) OR (mild cognitive impairment[Title/Abstract])) OR (MCI[Title/Abstract])) OR (memory[Title/Abstract])) OR (attention[Title/Abstract])) OR (executive function[Title/Abstract])) OR (processing speed[Title/Abstract])) AND ((((((((((((((((stroke[MeSH Terms]) OR (post-stroke cognitive impairment[Title/Abstract])) OR (PSCI[Title/Abstract])) OR (dementia, vascular[MeSH Terms])) OR (vascular dementia[Title/Abstract])) OR (vascular dementias[Title/Abstract])) OR (cerebral infarction[MeSH Terms])) OR (subcortical infarction[Title/Abstract])) OR (cerebral small vessel disease[Title/Abstract])) OR (CSVD[Title/Abstract])) OR (cerebrovascular disorders[MeSH Terms])) OR (brain ischemia[MeSH Terms])) OR (multi-infarct[Title/Abstract])) OR (cerebrovascular accident[Title/Abstract])) OR (vascular cognitive impairment[Title/Abstract])) OR (VCI[Title/Abstract])))

Web of Science

#1: (((((((TS=("brain-computer*")) OR TS=(BCI)) OR TS=("brain-machine*")) OR TS=(BMI)) OR TS=("neural interface")) OR TS=(neurofeedback)) OR TS=(electroencephalography)) OR TS=(EEG)

#2: (((((((((((((TS=("vascular cognitive impairment")) OR TS=(VCI)) OR TS=(stroke)) OR TS=("post-stroke cognitive impairment")) OR TS=(PSCI)) OR TS=("vascular dementia")) OR TS=("cerebral infarction")) OR TS=("subcortical infarction")) OR TS=("cerebral small vessel disease")) OR TS=(CSVD)) OR TS=("cerebrovascular disorders")) OR TS=("brain ischemia")) OR TS=(multi-infarct)) OR TS=("cerebrovascular accident")

#3: (((((((((((TS=("cogniti*")) OR TS=("cognitive dysfunction")) OR TS=("cognitive impairment")) OR TS=("cognitive disorder")) OR TS=("cognitive decline")) OR TS=("cognitive function")) OR TS=("mild cognitive impairment")) OR TS=(MCI)) OR TS=(memory)) OR TS=(attention)) OR TS=("executive function")) OR TS=("processing speed")

#1 AND #2 AND #3

Medline (via the Web of Science platform)

#1: (((((((TS=("brain-computer*")) OR TS=(BCI)) OR TS=("brain-machine*")) OR TS=(BMI)) OR TS=("neural interface")) OR TS=(neurofeedback)) OR TS=(electroencephalography)) OR TS=(EEG)

#2: (((((((((((((TS=("vascular cognitive impairment")) OR TS=(VCI)) OR TS=(stroke)) OR TS=("post-stroke cognitive impairment")) OR TS=(PSCI)) OR TS=("vascular dementia")) OR TS=("cerebral infarction")) OR TS=("subcortical infarction")) OR TS=("cerebral small vessel

disease")) OR TS=(CSVD)) OR TS=("cerebrovascular disorders")) OR TS=("brain ischemia")) OR TS=(multi-infarct)) OR TS=("cerebrovascular accident")

#3: ((((((((((TS=("cogniti*")) OR TS=("cognitive dysfunction")) OR TS=("cognitive impairment")) OR TS=("cognitive disorder")) OR TS=("cognitive decline")) OR TS=("cognitive function")) OR TS=("mild cognitive impairment")) OR TS=(MCI)) OR TS=(memory)) OR TS=(attention)) OR TS=("executive function")) OR TS=("processing speed")

#1 AND #2 AND #3

Embase

#1: 'brain computer interface'/exp OR 'brain computer interfaces':ab,kw,ti OR 'brain machine interface':ab,kw,ti OR 'brain machine interfaces':ab,kw,ti OR 'brain-computer interface':ab,kw,ti OR 'brain-computer interfaces':ab,kw,ti OR 'brain-machine interface':ab,kw,ti OR 'brain-machine interfaces':ab,kw,ti OR 'neurofeedback'/exp OR 'eeg biofeedback':ab,kw,ti OR 'eeg feedback':ab,kw,ti OR 'electroencephalography biofeedback':ab,kw,ti OR 'neurobiofeedback':ab,kw,ti

#2: 'mild cognitive impairment'/exp OR 'cognitive defect'/exp OR 'cognitive disorder':ab,kw,ti OR 'cognitive decline':ab,kw,ti OR 'cognitive deficiency':ab,kw,ti OR 'cognitive deficit':ab,kw,ti OR 'cognitive difficulties':ab,kw,ti OR 'cognitive disability':ab,kw,ti OR 'cognitive disturbance':ab,kw,ti OR 'cognitive dysfunction':ab,kw,ti OR 'cognitive impairment':ab,kw,ti OR 'memory'/exp OR 'attention'/exp OR 'attentiveness':ab,kw,ti OR 'processing speed'/exp OR 'executive function'/exp

#3: 'cerebrovascular accident'/exp OR 'cerebrovascular disease'/exp OR 'cerebrovascular disorders':ab,kw,ti OR 'multiinfarct dementia'/exp OR 'vascular dementia':ab,kw,ti OR 'brain infarction'/exp OR 'brain ischemia'/exp OR 'vascular cognitive impairment':ab,kw,ti OR 'stroke':ab,kw,ti OR 'post-stroke cognitive impairment':ab,kw,ti OR 'cerebral small vessel disease':ab,kw,ti

#1 AND #2 AND #3

Cochrane Central Register of Controlled Trials

#1: MeSH descriptor: [Brain-Computer Interfaces] explode all trees

#2: MeSH descriptor: [Neurofeedback] explode all trees

#3: ((brain-computer interface) OR (BCI) OR (BMI) OR (brain-machine interface) OR (neural interface) OR (electroencephalography) OR (EEG)):ti,ab,kw

#4: #1 OR #2 OR #3

#5: MeSH descriptor: [Cognitive Dysfunction] explode all trees

#6: ((cogniti*) OR (cognitive impairment) OR (cognitive disorder) OR (cognitive decline) OR (cognitive function) OR (mild cognitive impairment) OR (MCI) OR (memory) OR (attention) OR (executive function) OR (processing speed)):ti,ab,kw

#7: #5 OR #6

#8: MeSH descriptor: [Dementia, Vascular] explode all trees

#9: MeSH descriptor: [Cerebral Infarction] explode all trees

#10: MeSH descriptor: [Cerebrovascular Disorders] explode all trees

#11: MeSH descriptor: [Brain Ischemia] explode all trees

#12: MeSH descriptor: [Stroke] explode all trees

#13: ((vascular cognitive impairment) OR (VCI) OR (post-stroke cognitive impairment) OR (PSCI) OR (vascular dementia) OR (subcortical infarction) OR (cerebral small vessel disease) OR (CSVD) OR (multi-infarct) OR (cerebrovascular accident)):ti,ab,kw

#14: #8 OR #9 OR #10 OR #11 OR #12 OR #13

#4 AND #7 AND #14
